# The effects of IL-1β stimulated human umbilical cord mesenchymal stem cells on polarization and apoptosis of macrophages in rheumatoid arthritis

**DOI:** 10.1038/s41598-023-37741-6

**Published:** 2023-06-30

**Authors:** Ying-Xuan Zeng, Kuang-Yi Chou, Jeng-Jong Hwang, Hwai-Shi Wang

**Affiliations:** 1 Institute of Anatomy and Cell Biology, School of Medicine, National Yang Ming Chiao Tung University, Peitou, Taipei, 112 Taiwan, ROC; 2 School of Nursing, National Taipei University of Nursing and Health Sciences, Taipei, Taiwan, ROC; 3 Department of Medical Imaging, Department of Medical Imaging and Radiological Sciences, Chung Shan Medical University Hospital, Chung Shan Medical University, Taichung, Taiwan, ROC

**Keywords:** Cell biology, Immunology, Stem cells, Rheumatology

## Abstract

Macrophages play an important role in the pathogenesis of rheumatoid arthritis (RA), in which the functions of pro-inflammatory macrophages (M1) and anti-inflammatory macrophages (M2) are different. Our previous studies have demonstrated that interleukin-1β (IL-1β) stimulated human umbilical cord mesenchymal stem cells (hUCMSCs) increase the expression of tumor necrosis factor-related apoptosis-inducing ligand (TRAIL) and initiate breast cancer cell apoptosis via ligand to death receptor 4 (DR4) and DR5. In this study, we examined the effect of IL-1β stimulated hUCMSCs (IL-1β-hUCMSCs) on immunoregulation of M1 and M2 macrophages in vitro and in the RA mouse model. The results showed that IL-1β-hUCMSCs increased macrophage polarization into M2 macrophages and enhanced apoptosis of M1 macrophages in vitro. Moreover, the intravenous injected IL-1β-hUCMSCs in RA mice rehabilitated the imbalance of M1/M2 ratio and thus demonstrated the potential to reduce inflammation in RA. This study advances our knowledge of the underlying immunoregulatory mechanisms involved in IL-1β-hUCMSCs to induce M1 macrophage apoptosis and promote the anti-inflammatory polarization of M2 macrophages and demonstrates the potential of IL-1β-hUCMSCs to reduce inflammation in RA.

## Introduction

Rheumatoid arthritis (RA) is a chronic autoimmune joint disease characterized by infiltration of inflammatory cells such as lymphocytes and macrophages into the joints and progressive damage to periarticular and joint structures^1^. Current clinical treatments include non-steroidal anti-inflammatory drugs (NSAIDs), etanercept-TNF receptor blockers, methotrexate immune system inhibitors, or anti-TNF monoclonal antibodies^2^. However, the efficacy of these treatments is not ideal. For example, anti-rheumatic drugs have many side effects, which may lead to problems such as gastrointestinal discomfort and liver damage^3^. Therefore, the development of new treatment modalities are urgent. Since macrophages are one of the inflammatory cell types in the RA synovium and they are involved in the pathogenesis of joint erosions in RA^4^, macrophages may be a new potential therapeutic target in RA. Given the potentially different functions of pro-inflammatory macrophages (M1) and anti-inflammatory macrophages (M2)^5,6^, it is also worthwhile to target treatments to specific types of macrophages.

Macrophages are important in innate immunity and are capable of polarizing into different types with diverse functions, such as M1 macrophages and M2 macrophages^7,8^. M1 macrophages are classically activated by IFN-γ or lipopolysaccharide (LPS) with inducible nitric oxide synthase (iNOS) being an important marker^9–11^. M2 macrophages are alternatively activated by exposure to IL-4^12,13^, IL-10 or IL-13^13^. CD163 is also a marker of alternatively activated M2 macrophages^14,15^. M1 macrophages produce pro-inflammatory factors and are responsible for inflammatory signaling. M2 macrophages produce anti-inflammatory cytokines and have tissue repair functions^13^. When tissue is damaged, macrophages are polarized into the M1 macrophages and secrete pro-inflammatory factors to fight the pathogen. Macrophages are then polarized to the M2 macrophages, secreting anti-inflammatory factors and performing tissue repair^16^. There is already evidence that many diseases are associated with an imbalance of M1 and M2 macrophages, as RA patients have higher M1/M2 ratios than normal^17^. It has been found that the anti-apoptotic ability of M1 macrophages in RA synovium is greater than that of M2 macrophages^18^. Enhancing the anti-apoptotic ability of M2 macrophages or increasing M2 macrophages may be an effective treatment for RA. Therefore, to strengthen the therapeutic efficacy for patients with active RA, it would be better to boost resident anti-inflammatory M2 macrophages or eliminate pro-inflammatory M1 macrophages.

Mesenchymal stem cells (MSCs) are easy to obtain without major ethical issues, and have low immunogenicity, which makes MSCs a possible therapeutic with enormous potential in different diseases^19^. Many studies indicate that MSCs can be used to deliver antitumor proteins such as interferons, interleukins, and soluble tumor necrosis factor-related apoptosis-inducing ligands (TRAIL)^20,21^. Human Umbilical Cord-derived Mesenchymal Stem Cells (hUCMSCs) possess paracrine effect and migration ability toward inflammation sites^22^ with beneficial therapeutic application in RA.

Tumor necrosis factor-related apoptosis-inducing ligand (TRAIL) is a type II transmembrane protein. It has been demonstrated that TRAIL can selectively induce apoptosis in various tumor cells in vitro^23,24^. TRAIL has five receptors^25^, of which death receptor 4 (DR4, TRAIL-R1) and death receptor 5 (DR5, TRAIL-R2) are associated with TRAIL-induced apoptosis upon binding^26,27^. TRAIL is involved in two pathways of apoptosis, extrinsic and intrinsic. TRAIL binds to DR4 or DR5, then initiates the extrinsic pathway by activating caspase 8 and caspase 3. Sometimes the intrinsic pathway is required for activated caspase 8, which eventually leads to cell death^28^. It has been found that administration of adenoviral vectors with TRAIL to rabbit knees not only induced apoptosis of synovial cells, but also reduced inflammation in a model of arthritis^29^.

Interleukin-1β (IL-1β), is one of the main mediators of the innate immune response and mediates inflammatory effects^30^. IL-1β-activated MSCs can regulate immune cell homeostasis^31^. Studies have pointed out that MSC exosomes initiated by IL-1β enhance anti-inflammatory activity in osteoarthritis cells^32^. In our previous study, we found that TRAIL expression in hUCMSCs can be upregulated after IL-1β stimulation. Thus, enhancing the apoptosis of M1 macrophages via IL-1β induced TRAIL expression hUCMSCs may decrease levels of pro-inflammatory cytokine secretion in RA.

The purpose of this study is to investigate the effects of IL-1β-stimulated hUCMSCs (IL-1β-hUCMSCs) on the polarization of macrophages and to examine the effects of IL-1β-hUCMSCs on apoptosis and proliferation of M1 and M2 macrophages. Moreover, the effect of IL-1β-hUCMSCs on the ratio of M1 and M2 macrophages of the RA mouse joints model was examined to confirm IL-1β-hUCMSCs as a potential treatment to target specific macrophages in RA.

## Results

### Cytotoxic impact of LPS and IL-4 on Raw264.7 cells and KG-1 cells

**Figure 1:**
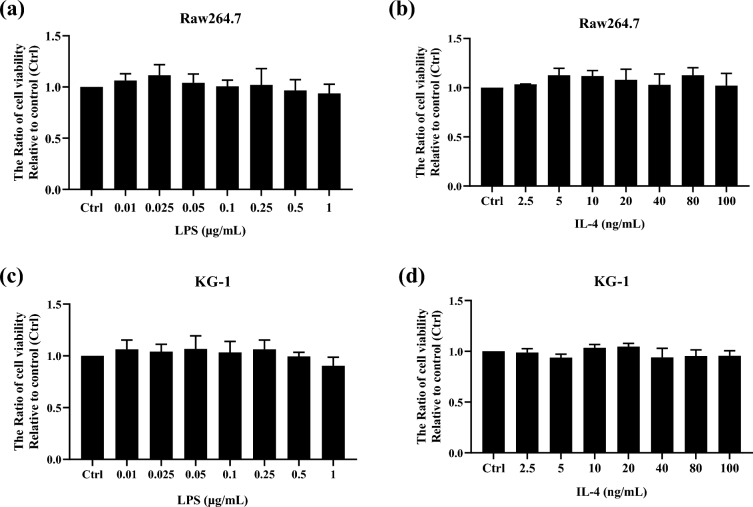
Cytotoxic impact of LPS and IL-4 on macrophage cells. (**a**, **b**) Raw264.7 and (**c**, **d**) KG-1 were treated with LPS at different concentrations (0.01, 0.025, 0.05, 0.1, 0.25, 0.5, 1 μg/mL) and IL-4 at different concentrations (2.5, 5, 10, 20, 40, 80, 100 ng/mL) for 24 h. Results were obtained using MTT assay. The quantified data was detected by multimode microplate readers at a wavelength of 545 nm. Statistical analysis data was analyzed by one-way ANOVA with Dunnett's test. Data are shown as mean ± SEM (n = 3).

To examine the cytotoxicity of Lipopolysaccharide (LPS) and Interleukin-4 (IL-4), Raw264.7 cells and KG-1 cells were treated with 0 to 1 μg/mL LPS and 0 to 100 ng/mL IL-4 for 24 h and detected by MTT assay. The results showed that the different concentrations of LPS and IL-4 used in these experiments had no significant cytotoxic effects on these two cell lines (Fig. 1a–d). Therefore, the concentration of 0.025, 0.5, 1 μg/mL LPS and 5, 10, 20 ng/mL IL-4 were used for the subsequent macrophage polarization experiments.

### The effects of LPS and IL-4 in macrophages polarization, DR4 and DR5 expression

**Figure 2:**
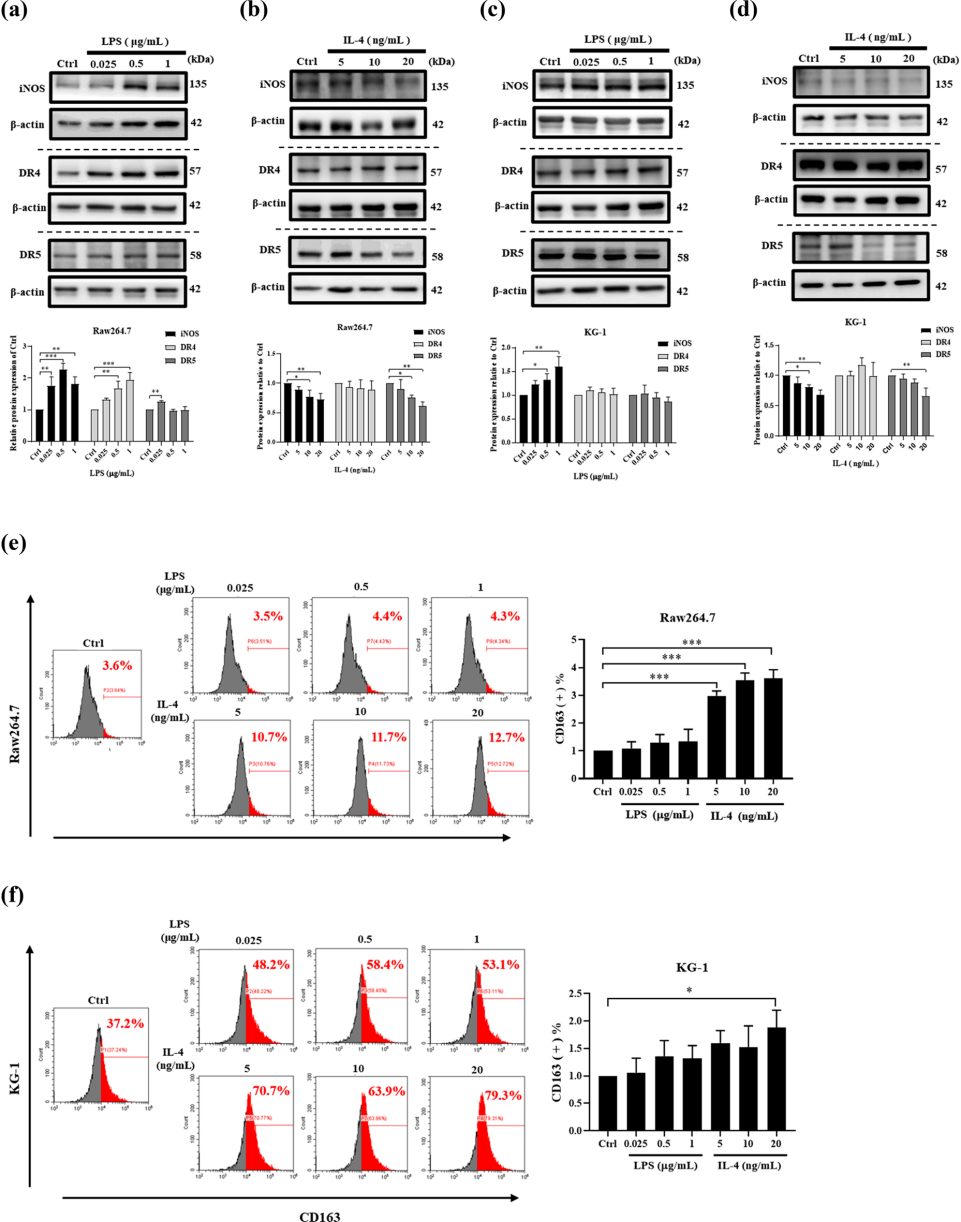
LPS and IL-4 induce macrophage cells polarization and expression of DR4 and DR5. Raw264.7 and KG-1 were induced with different doses of LPS (0.025, 0.5, 1 μg/mL) and IL-4 (0, 5, 10, 20 ng/mL) for 24 h. The expression of iNOS, DR4 and DR5 in (**a**, **b**) Raw264.7 cells and (**c**, **d**) KG-1 cells were detected by western blot and quantified by using AlphaEaseFC 4.0 software. The full-length Western blots were shown in Supplementary Fig. S1. Flow cytometry was used to analyze expression of CD163 in Raw264.7 cells (**e**) and KG-1 cells (**f**). Statistical analysis data was analyzed by one-way ANOVA with Dunnett's test (n = 3). Data are shown as mean ± SEM (n = 3, *P < 0.05, **P < 0.01, ***P < 0.001).

Western blot and flow cytometry were used to examine whether LPS and IL-4 induced Raw264.7 cells and KG-1 cells polarized into M1 macrophages or M2 macrophages. After treatment with LPS or IL-4, the expression of DR4 and DR5 in both cell lines were also detected. The Western blot data showed that treatment with 0.5 and 1 μg/mL LPS significantly increased the expression of iNOS in Raw264.7 cells (Fig. 2a) and in KG-1 cells (Fig. 2c). DR4 and DR5 expression only increased in LPS induced Raw264.7 cells. DR4 expression increased after treatment with 0.5 and 1 μg/mL LPS and DR5 increased after treatment with 0.025 μg/mL LPS (Fig. 2a). The results indicated that macrophages with LPS treatment were successfully polarized to M1 macrophages and DR4 and DR5 expressions were higher in these M1-polarized macrophages. Treatment with 10 and 20 ng/mL IL-4 decreased the expression of iNOS in Raw 264.7 cells (Fig. 2b) and KG-1 cells (Fig. 2d). The expression of DR5 was decreased in both 20 ng/mL IL-4 induced Raw264.7 cells (Fig. 2c) and KG-1 cells (Fig. 2d).

The M2 phenotype status of LPS and IL-4 induced Raw 264.7 cells and KG-1 cells were characterized by CD163 expression via flow cytometry. The results showed that the expression of CD163 in Raw 264.7 cells and KG-1 did not increase after being treated with LPS (Fig. 2e,f). However, the expression of CD163 significantly increased in 5, 10, and 20 ng/mL IL-4 stimulated Raw 264.7 cells (Fig. 2e) and in 20 ng/mL IL-4-stimulated KG-1 cells (Fig. 2f). According to the above experimental data, 1 μg/mL LPS and 20 ng/mL IL-4 were chosen for use in the subsequent experiments. These results demonstrated that LPS could induce M1 polarization and IL-4 could successfully induce M2 polarization in Raw 264.7 cells and KG-1 cells. Moreover, TRAIL receptors DR4 and DR5 expression significantly increased in LPS induced M1 polarized Raw 264.7 cells. DR5 expression was decreased in IL-4 treated Raw 264.7 cells and KG-1 cells, suggesting that DR5 expression was decreased in IL-4 induced M2 polarization in Raw 264.7 cells and KG-1 cells.

### The effect of IL-1β-hUCMSCs on macrophages polarization

**Figure 3:**
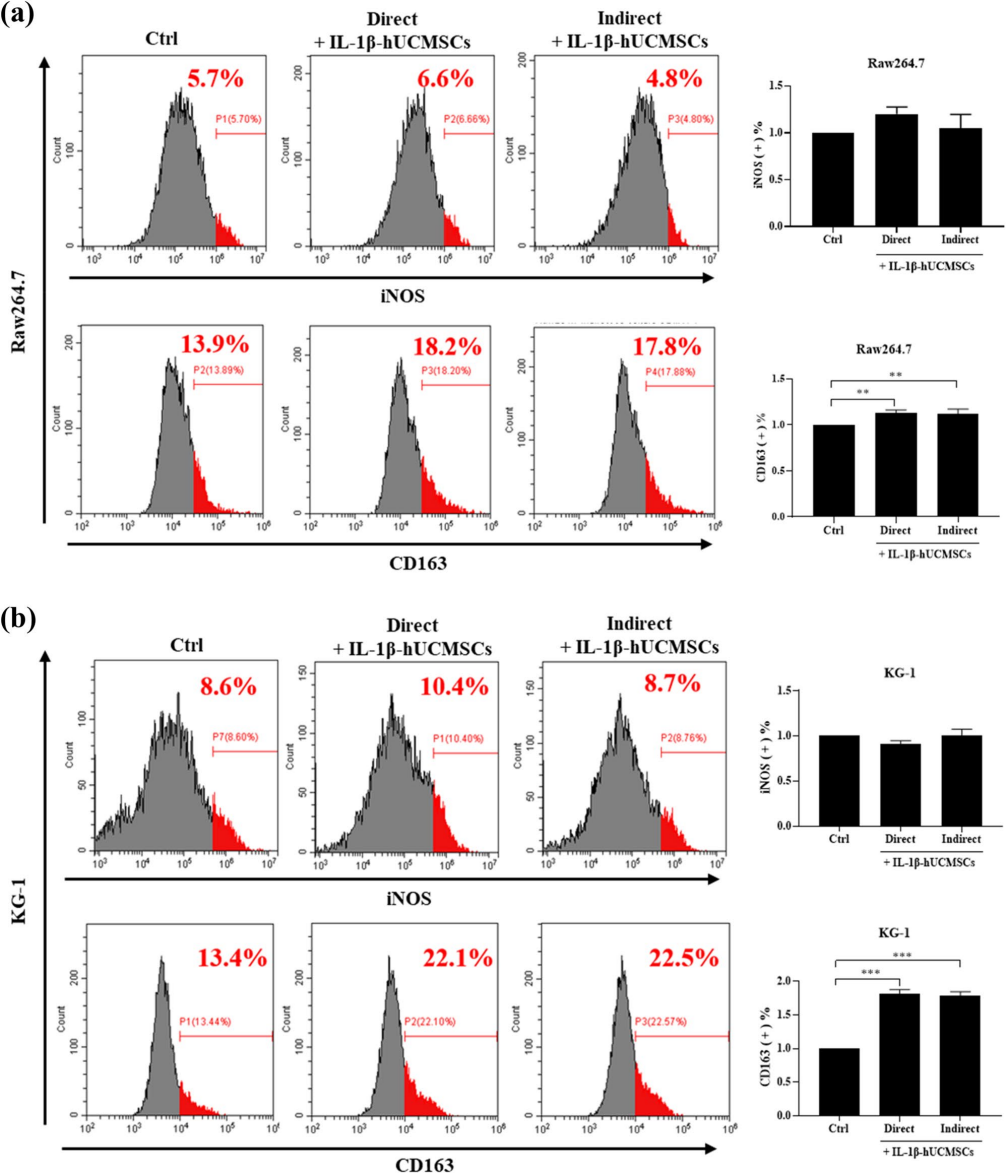
IL-1β-hUCMSCs polarize Raw264.7 and KG-1 into M2 macrophages. IL-1β-hUCMSCs (IL-1β-hUCMSCs) were directly co-cultured or indirectly co-cultured with Raw264.7 and KG-1 for 24 h. After co-culturing, cells were detected by flow cytometry to analyze the expression of iNOS and CD163 in (**a**) Raw264.7 cells and (**b**) KG-1 cells. Statistical analysis data was analyzed by one-way ANOVA with Dunnett's test. Data are shown as mean ± SEM (n = 3, **P < 0.01, ***P < 0.001).

Macrophage infiltration is one of the symptoms in rheumatoid arthritis^27^. A large number of M1 macrophages secrete pro-inflammatory factors, which not only aggravates the disease but also makes more M0 macrophages polarized into M1 macrophages. To know whether IL-1β-hUCMSCs could induce macrophage polarization, we co-cultured macrophages with IL-1β-hUCMSCs directly or indirectly. Flow cytometry data showed that the expression of iNOS was not statistically significantly different in Raw264.7 and KG-1 cells directly or indirectly co-cultured with IL-1β-hUCMSCs. However, the expression of CD163 in both Raw264.7 and KG-1 cells significantly increased after direct and indirect co-culture with IL-1β-hUCMSCs (Fig. 3a,b). The results show that Raw 264.7 cells and KG-1 cells co-cultured with IL-1β-hUCMSCs did not increase M1 macrophages polarization but increased M2 macrophages polarization.

### IL-1β-hUCMSCs induce the apoptosis of M1 macrophage

**Figure 4:**
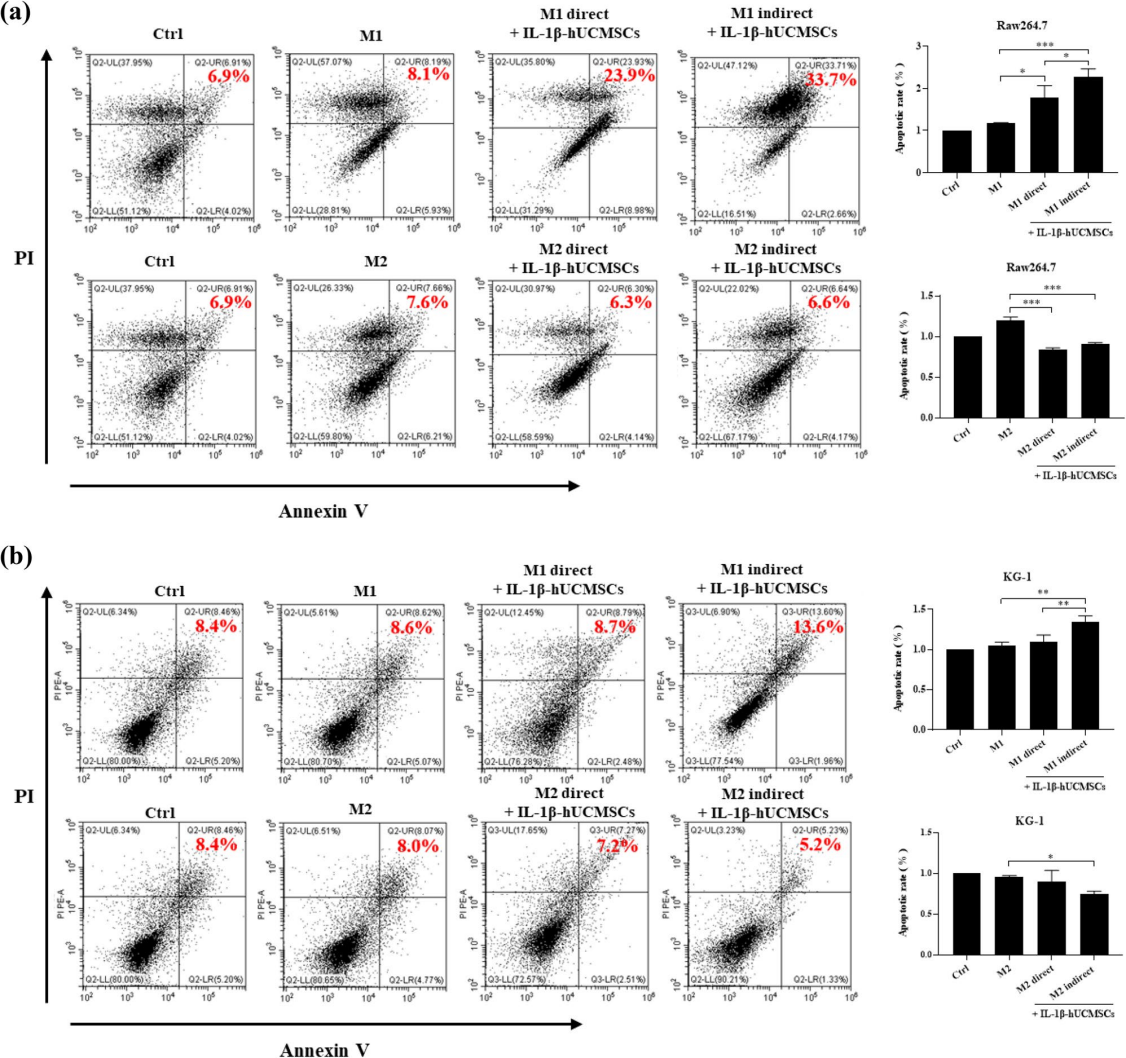
IL-1β-hUCMSCs induce M1 macrophages apoptosis in Raw264.7 cells and KG-1 cells. Raw264.7 and KG-1 cells were treated 1 µg/mL LPS or 20 ng/mL IL-4 for 24 h to induce into M1 or M2 macrophages, then co-cultured with IL-1β-hUCMSCs for 24 h. After co-culturing, cells were labeled with CD105 antibody to distinguish hUCMSCs from macrophages and stained with Annexin V-FITC and PI to analyze the apoptosis of (**a**) Raw264.7 cells and (**b**) KG-1 cells. Statistical analysis data was analyzed by one-way ANOVA with Tukey's test. Data are shown as mean ± SEM (n = 3, *P < 0.05, **P < 0.01, ***P < 0.001).

Aggregation of M1 macrophages have been found to promote inflammation in rheumatoid arthritis^33^. Therefore, we wanted to know the effects of IL-1β-hUCMSCs on apoptosis of M1 macrophages. Raw264.7 cells and KG-1 cells were induced into M1 and M2 macrophages then directly or indirectly co-cultured with IL-1β-hUCMSCs. Flow cytometry data showed that the apoptotic rate of M1-polarized Raw264.7 cells was increased after direct and indirect co-culture with IL-1β-hUCMSCs (Fig. 4a), while the apoptotic rate of M2-polarized Raw264.7 cells was decreased (Fig. 4a). The apoptotic rate of M1-polarized KG-1 cells significantly increased after indirect co-cultured with IL-1β-hUCMSCs (Fig. 4b). However, the apoptotic rate of M2-polarized Raw264.7 cells and KG-1 cells decreased after indirect co-culture with IL-1β-hUCMSCs (Fig. 4a,b). These data suggested that IL-1β-hUCMSCs could enhance the apoptosis of M1 macrophages and inhibit the apoptosis of M2 macrophages.

### IL-1β-hUCMSCs induced M1 macrophages apoptosis via extrinsic and intrinsic apoptotic pathways

**Figure 5:**
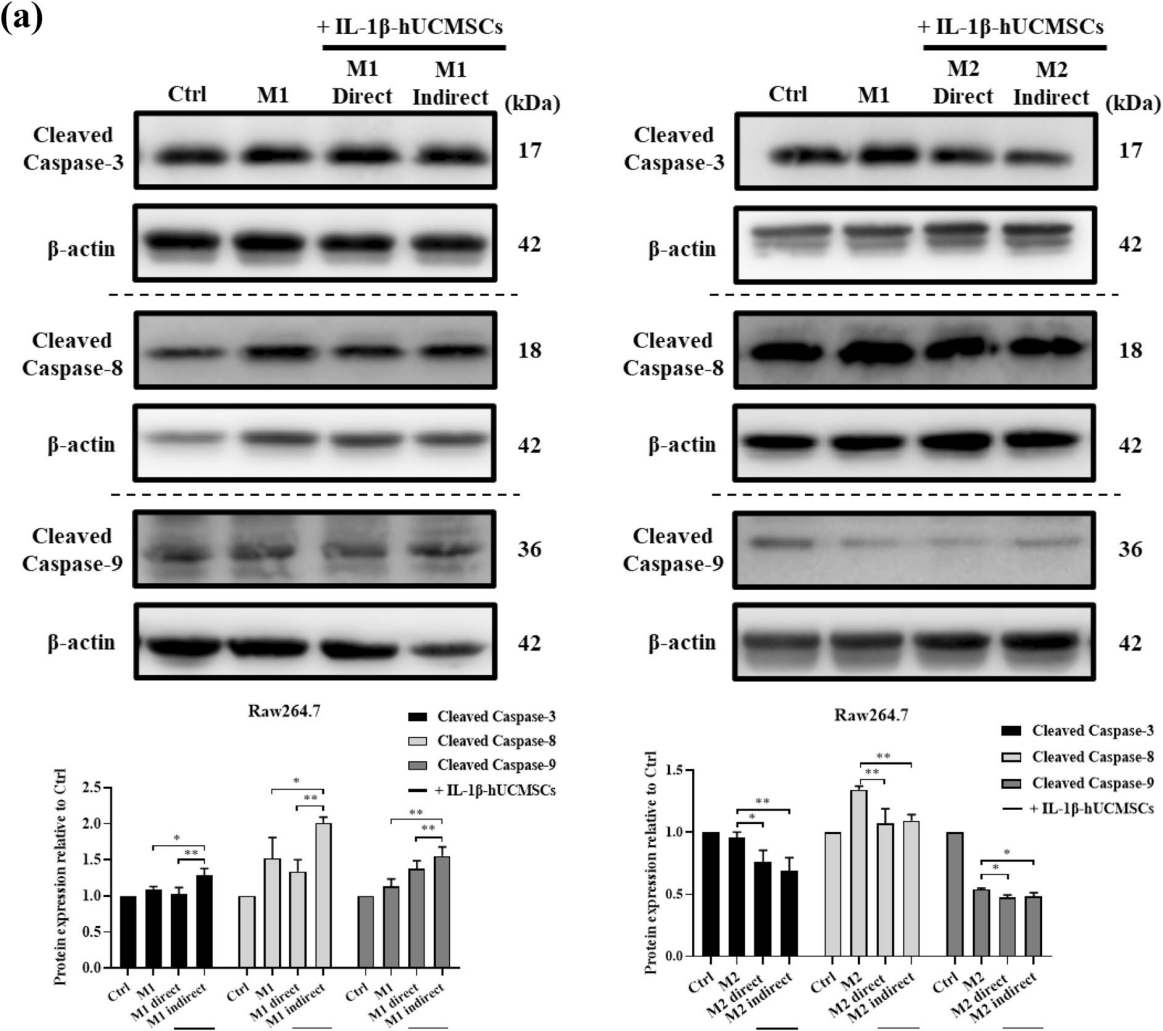

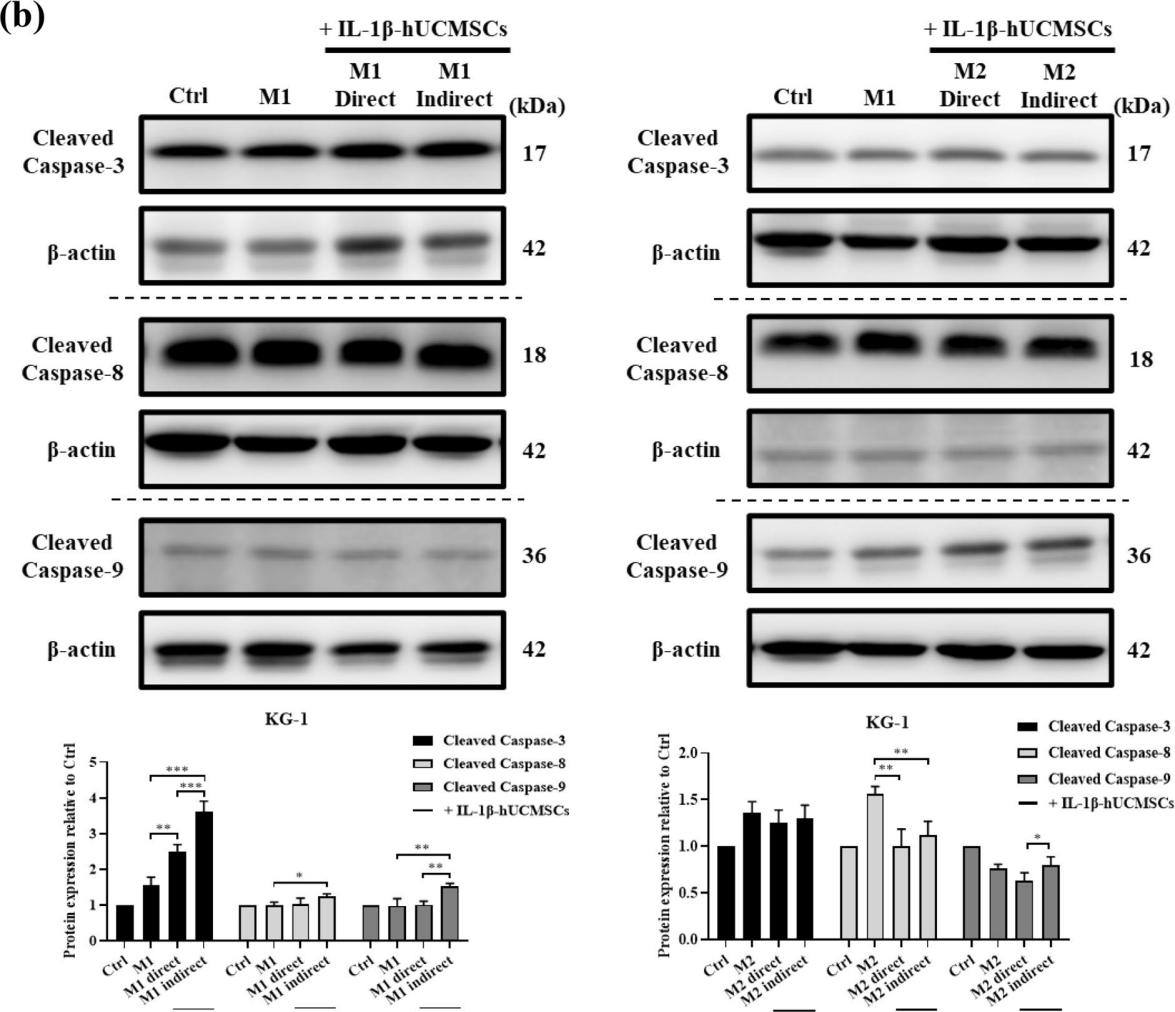
IL-1β pre-stimulated hUCMSCs induce M1 macrophages to utilize extrinsic and intrinsic pathways for apoptosis (**a**) Raw264.7 cells and (**b**) KG-1 cells were induced by 1 μg/mL LPS and 20 ng/mL IL-4 to M1 macrophages and M2 macrophages, and then co-cultured with IL-1β-hUCMSCs for 24 h. The expression of cleaved caspase-3, -8 and -9 was detected by western blot and quantified using AlphaEaseFC 4.0 software. The full-length Western blots were shown in Supplementary Fig. S1. Statistical analysis data was analyzed by one-way ANOVA with Tukey's test. Data are shown as mean ± SEM (n = 3, *P < 0.05, **P < 0.01, ***P < 0.001).

The two main pathways of apoptosis are extrinsic and intrinsic pathways^34^. To determine which apoptotic pathway is involved in IL-1β-hUCMSCs-induced M1 macrophage apoptosis, the expression of cleaved caspase-3, -8, and -9 was detected by Western blot. Compared with the M1-polarized cells group, the results showed that the expressions of cleaved caspase-3, -8, and -9 were significantly increased in the groups of M1-polarized Raw264.7 cells and KG-1 cells indirect co-cultured with IL-1β-hUCMSCs (Fig. 5a,b). In M2-polarized Raw264.7 cells, the expression of cleaved caspase-3, -8 and -9 significantly decreased after indirect co-cultured with IL-1β-hUCMSCs (Fig. 5a). But only cleaved caspase-8 significantly decreased in M2-polarized KG-1 cells indirectly co-cultured with IL-1β-hUCMSCs (Fig. 5b). However, compared with the M2-polarized KG-1 cells, the expression of cleaved caspase-3 and -9 had no significant difference after direct or indirect co-culture with IL-1β-hUCMSCs (Fig. 5b). The results suggested that both extrinsic and intrinsic apoptotic pathways are involved in IL-1β-hUCMSCs-induced M1 macrophages apoptosis.

### The effects of IL-1β-hUCMSCs on macrophage proliferation

**Figure 6:**
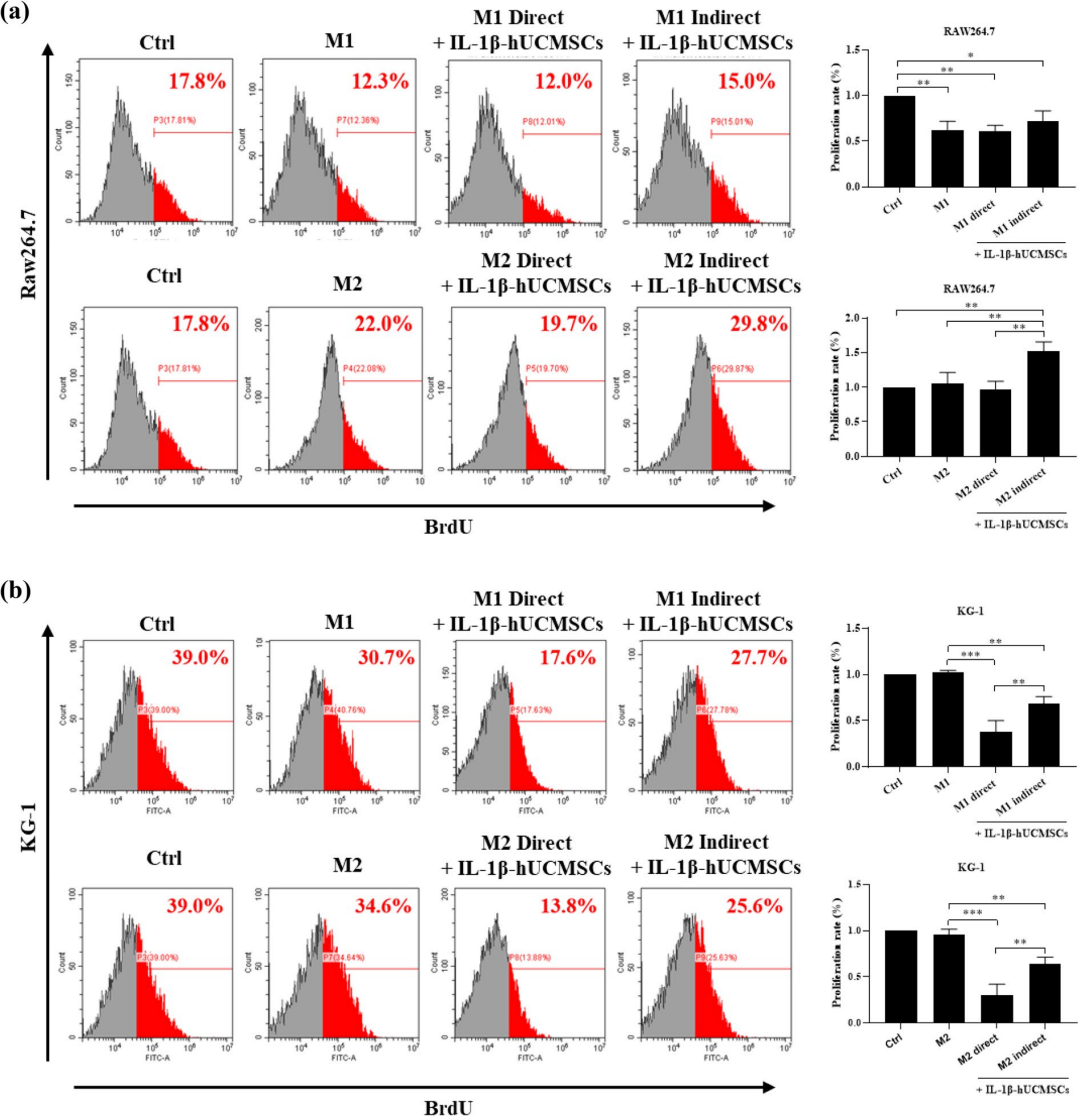
IL-1β-hUCMSCs decrease the proliferation of M1-polarized Raw264.7 cells and KG-1 cells. M1, M2-polarized (**a**) Raw264.7 cells and (**b**) KG-1 cells were co-cultured with IL-1β-hUCMSCs for 24 h and treated with 10 µM BrdU for an hour. The cell proliferation was detected by Flow cytometry. Statistical analysis data was analyzed by one-way ANOVA with Tukey's test. Data are shown as mean ± SEM (n = 3, *P < 0.05, **P < 0.01, ***P < 0.001).

We had demonstrated that the apoptotic rate of M1 macrophages increased after co-culture with IL-1β-hUCMSCs. Next, we examine the effect of IL-1β-hUCMSCs on M1 and M2 macrophage proliferation. The results of flow cytometry showed that IL-1β-hUCMSCs had no effect on the proliferation of M1-polarized Raw246.7 cells. But increased M2-polarized Raw246.7 cells proliferation after indirect co-culture with IL-1β-hUCMSCs (Fig. 6a). While the proliferation rate of M1-polarized and M2-polarized KG-1 cells decreased after co-culture with IL-1β-hUCMSCs (Fig. 6b). According to the above results, the effects of IL-1β-hUCMSCs on macrophage proliferation might be related to increased M1 macrophage cell apoptosis and M2 macrophage proliferation.

### The ratio of M1 and M2 macrophages in synovial tissues of CIA mice after intravenous injection of IL-1β-hUCMSCs

**Figure 7:**
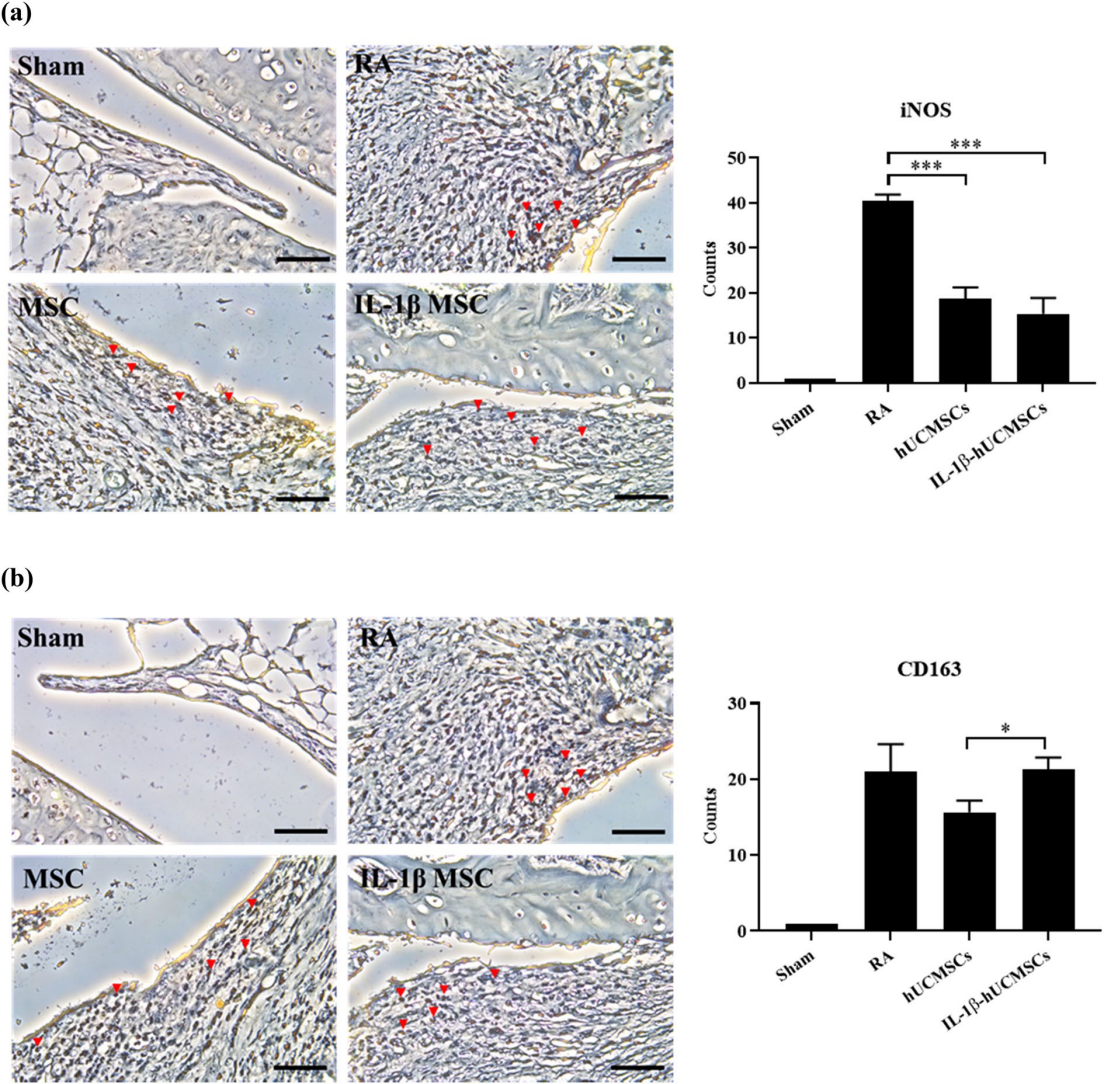
The M1 and M2 macrophages characterization staining in RA mice joint tissues. CIA mice were sacrificed on day 20 with or without IL-1β-hUCMSCs administration. The sections were stained with (**a**) M1 macrophage marker iNOS antibody and (**b**) M2 macrophage marker CD163 antibody. Graph indicates the number of the iNOS positive cells (M1 macrophages) (**a**) and CD163 positive cells (M2 macrophages) (**b**). Macrophages are marked with arrowheads. Scale bar: 50 μm. Statistical analysis data was analyzed by one-way ANOVA with Tukey's test. Data are shown as mean ± SEM (n = 3, *P < 0.05, ***P < 0.001).

The effect of IL-1β-hUCMSCs in the CIA mouse model was examined. Mice were induced to develop RA then injected with IL-β-hUCMSCs by intravenous injection and sacrificed after 20 days to obtain the articulating tissue. Immunohistochemical staining was performed to examine the presence of M1 and M2 macrophages in the joint sections. The results showed that the RA group had more M1 macrophages, but the number of M1 macrophages decreased after injection of IL-1β-hUCMSCs (Fig. 7a). However, compared with the MSC group, more M2 macrophages can be found in the sections of the IL-1β-hUCMSCs group (Fig. 7b). By analyzing the M1/M2 ratio, we found that the ratio was 1.9 in the RA group, 1.2 in hUCMSCs group, and the ratio was decreased to 0.7 in IL-1β-hUCMSCs group. This result demonstrates the significant effect of IL-1β-hUCMSCs treatment in modulating M2 macrophage polarization as compared to using hUCMSCs alone.

## Discussion

Rheumatoid arthritis is a chronic systemic autoimmune disease that affects the joints of the patients. Monocytes and macrophages are at the core of RA inflammation, and the expression of inflammatory M1 macrophages is increased in RA^35^. Even though there are many RA treatments available, the therapies for RA sometimes fail or are only partially successful^36^. Therefore, in recent years, the prevention or reduction of inflammatory macrophages to alleviate symptoms is now part of the research direction, as a new target therapy in RA.

To ameliorate RA, some macrophage-targeted treatments have been applied, however, these therapies do not specifically target pro-inflammatory macrophages. A strategy for RA therapy would be to develop treatments that prevent the reduction of anti-inflammatory macrophages or aim at impeding the hyper-infiltration of pro-inflammatory macrophages in the RA joint synovium. Lipopolysaccharide (LPS) can induce macrophage polarization to the M1 macrophages and interleukin 4 (IL-4) can drive macrophage polarization to M2 macrophages^37^. In this study, we used LPS and IL-4 to induce macrophage to M1 and M2 macrophages to investigate the mechanisms underlying the therapeutic effects of IL-1β-hUCMSCs on macrophage polarization and macrophage apoptosis in rheumatoid arthritis.

TRAIL is a pro-apoptotic protein that can induce malignant cell death, but different cells have different sensitivity to TRAIL, making it difficult to use TRAIL as a therapy. In our previous study, we found that both membrane bound TRAIL and soluble TRAIL expression in hUCMSCs can be upregulated after IL-1β stimulation. By binding to DR4 and DR5,

TRAIL initiates apoptotic pathway^38^. Many cytokines and drugs can enhance TRAIL-mediated apoptosis by upregulating DR4 and DR5 expressions. In this study, we found that the increase of TRAIL in IL-1β-hUCMSCs can enhance M1 macrophages apoptosis (Fig. 4) via the up-regulation of cleaved caspase-3, cleaved caspase-8, and cleaved caspase-9, which confirms that TRAIL triggers M1 macrophage apoptosis through extrinsic and intrinsic pathways (Fig. 5).

In rheumatoid arthritis, the polarization of macrophages plays a crucial role. M1 macrophages produce pro-inflammatory factors, aggravate the symptoms of RA, and cause joint and periarticular damage, while M2 macrophages release anti-inflammatory factors to repair the symptoms of RA^39,40^. Because of the different underlying functions of M1 and M2 macrophages, it is best to reduce M1 macrophages or increase M2 macrophages to ameliorate RA symptoms. The application of monoclonal antibodies against macrophage-colony stimulating factor (M-CSF) has been found to reduce the number of monocytes in RA patients^37^. Sinomenine, the main active ingredient of the plant Sinomenium acutum, can regulate the secretion of inflammatory cytokines and monocyte or macrophage subsets^41^. However, the target and underlying mechanism of sinomenine to suppress RA progression has not been elucidated. In our experiments, we demonstrated that IL-1β-hUCMSCs could slow the proliferation of M1 macrophages (Fig. 6), and IL-1β-hUCMSCs could also increase the rate of macrophage polarized into M2 macrophages. The summarized results in Table 1 show that the apoptosis rate was increased under direct and indirect co-culture with IL-1β-hUCMSCs. M1 macrophages indirectly co-cultured with IL-1β-hUCMSCs showed more impact in increasing the apoptosis rates and the expression levels of cleaved caspase-3, -8, -9 (Table 1). From our previous study, we found that both soluble and membrane bound TRAIL can be induced by IL-1β stimulation^42^. Thus, the action of soluble TRAIL present in the indirect co-culture conditioned medium may be more effective in inducing M1 macrophage apoptosis. On the contrary, the proliferation of M1 and M2 macrophages decreased in direct co-culture conditions in comparison with indirect co-culture with IL-1β-hUCMSC (Table 1). This effect may involve cell-to-cell contact between macrophages and IL-1β-hUCMSCs, leading to direct interactions and signal transductions that could have a more pronounced effect on macrophage proliferation.

**Table 1:** Summary of the results of apoptosis rates, expression levels of cleaved caspase-3, -8, -9, and proliferation rates of M1 and M2 polarized Raw264.7 and KG-1 cells directly or indirectly co-cultured with IL-1β-hUCMSCs.

	M1	M1 + IL-1β-hUCMSC (direct)	M1 + IL-1β-hUCMSC (indirect)	M2	M2 + IL-1β-hUCMSC (direct)	M2 + IL-1β-hUCMSC (indirect)
Raw264.7						
Apoptosis rate	/	+	+++	/	− − −	− − −
Cleaved caspase 3	/	NS	+	/	−	− −
Cleaved caspase 8	/	NS	+	/	− −	− −
Cleaved caspase 9	/	NS	++	/	−	−
Proliferation rate	− −	− −	−	NS	NS	++
KG-1						
Apoptosis rate	/	NS	++	/	NS	−
Cleaved caspase 3	/	++	+++	/	NS	NS
Cleaved caspase 8	/	NS	+	/	− −	− −
Cleaved caspase 9	/	NS	++	/	NS	NS
Proliferation rate	NS	− − −	− −	NS	− − −	− −

/ no comparison, *NS* no significant difference, − decrease, + increase.

**Figure 8:**
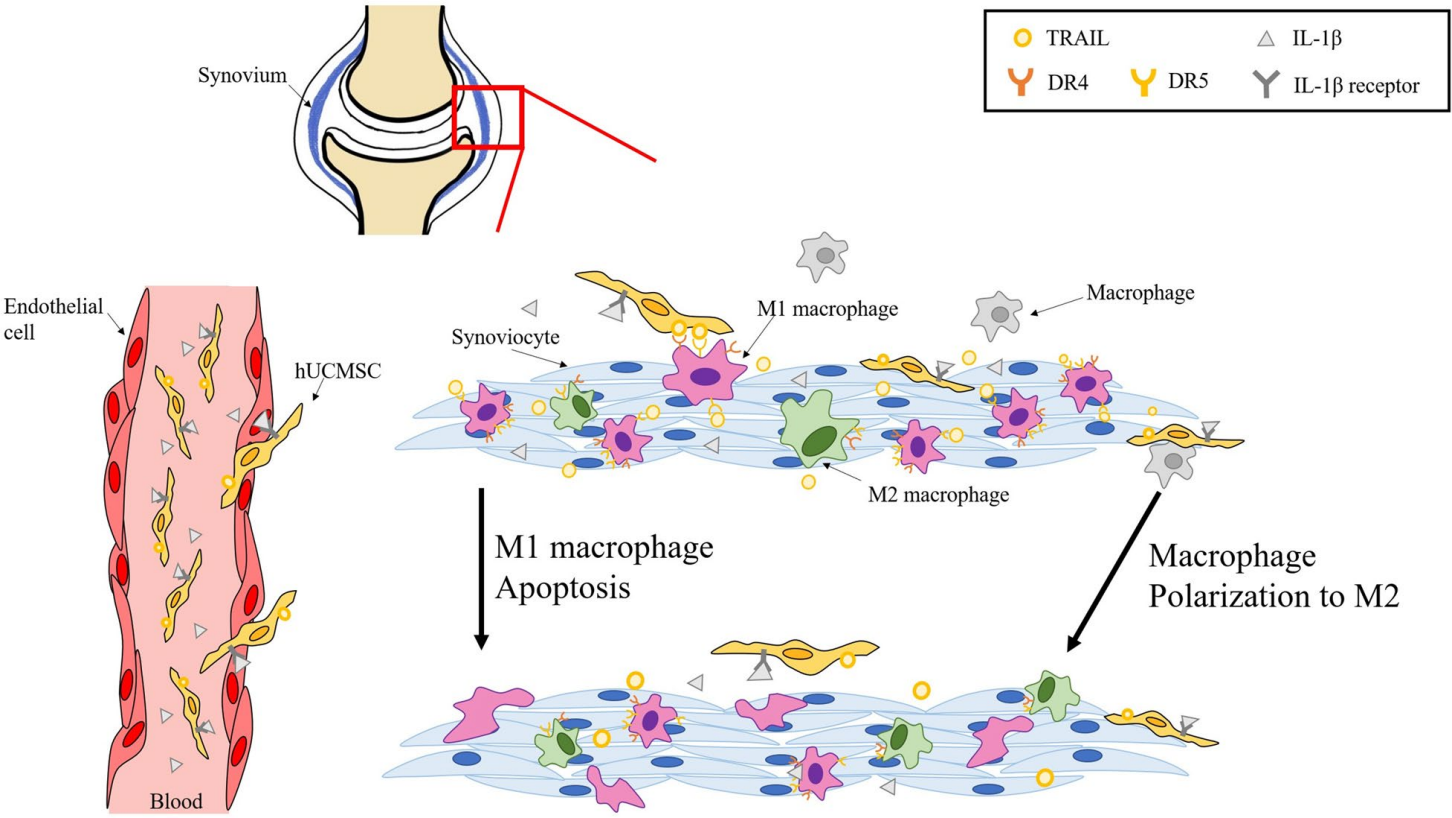
Schematic diagram of IL-1β-hUCMSCs inducing M1 macrophage apoptosis and promoting macrophage polarization into M2 macrophage in RA synovium. A schematic diagram depicting IL-1β-hUCMSCs homing to inflammation sites to induce M1 macrophage apoptosis via TRAIL-DR4/5. Moreover, IL-1β-hUCMSCs promote macrophage polarization into M2 macrophages in RA synovium.

According to our results, after successfully inducing macrophages into M1 macrophages and M2 macrophages, TRAIL secreted by IL-1β-hUCMSCs can induce apoptosis of M1 macrophages through both internal and external pathways and reduce their ability to proliferate. In addition, IL-1β-hUCMSCs promote M2 macrophage polarization. In RA synovial tissues, the proportion of pro-inflammatory M1 macrophages is higher than anti-inflammatory M2 macrophages. Targeting M1 macrophages and enhancing the polarization of M2 macrophages in RA to rebalance the M1/M2 ratio would be suitable for RA therapy. MSCs have been used as a promising therapy for immune-related diseases^43,44^. Results from an in vivo mouse model demonstrated that synovial M1 macrophages were reduced and M2 macrophages were increased after injection of IL-1β-hUCMSCs (Fig. 7). Compared with the hUCMSCs group, the M1/M2 ratio of the IL-1β-hUCMSCs group supports IL-1β-hUCMSCs as a potential therapeutic strategy. The results of this in vivo study are consistent with the in vitro data that IL-1β-hUCMSCs induce apoptosis of M1 macrophages via TRAIL/DR4 or TRAIL/DR5 interaction and enhance M2 macrophage polarization. In summary, this study indicates that the expression of DR4 and DR5 were upregulated in M1 macrophages. TRAIL expression in hUCMSCs was enhanced after IL-1β stimulation. Via TRAIL/DR4/5 interaction, IL-1β-hUCMSCs induce the apoptosis of M1 macrophages. Furthermore, IL-1β-hUCMSCs enhance the polarization of M2 macrophages (Fig. 8).

We conclude that this study advances our understanding of the immunoregulatory mechanisms involved in intravenous injected IL-1β-hUCMSCs in a RA mice model and hints at the potential of hUCMSCs for RA therapy. However, several limitations in this study that could be addressed in future research include determining the mechanisms and signaling pathways involved in IL-1β-hUCMSCs enhanced M2 polarization. Whether the rehabilitated M1/M2 ratio could affect the synovial hyperplasia in RA synovial intima is also worthy of investigation. The effects of IL-1β-hUCMSCs in this RA model were observed after administration of injections for 20 days. Thus, the issues of long-term efficacy and safety of IL-1β-hUCMSCs treatment in RA therapy still needs to be studied.

## Methods

### Human umbilical cord mesenchymal stem cell (hUCMSCs)

Human umbilical cord mesenchymal stem cells were acquired from the Bioresource Collection and Research Center, Hsinchu, Taiwan. Cells were cultured in medium which contain 56% Low-glucose Dulbecco's Modified Eagle Medium (DMEM-LG; Life technology, NY, USA), 37% MCDB201 Medium (Sigma, MO, USA), 2% fetal bovine serum (Thermo, Logan, UT), 0.5 mg/mL of bovine serum albumin fraction V (Sigma, MO, USA), 10 ng/mL epidermal growth factor (PeproTech, NJ, USA), 1 ng/mL of platelet-derived growth factor-BB (PeproTech, NJ, USA), 50 μM l-Ascorbic acid 2-phosphate sesquimagnesium salt hydrate (Sigma, MO, USA), 10 nM Dexamethasone (Sigma, MO, USA), 1 × antibiotic–antimycotic solution (Corning, NY, USA), 1 × Insulin-Transferrin-Selenium-A (Life technology, NY, USA), and sterilized secondary water to total volume. The cells were incubated at 37 °C and 5% CO_2_. Cells were separated by Accutase ® (Innovative Cell Technologies, SD, USA) when cells reached confluency of 80% density and reseeded 8 × 10^5^ cells per 100 mm cell culture dish.

### Macrophage cell lines and cell culture

The RAW264.7 cells are macrophage-like cells derived from mice. The KG-1 cell line is made up of macrophages isolated from the bone marrow of a human. Both cell lines were purchased from ATCC, USA. The Raw264.7 cells were cultured in High-glucose Dulbecco's Modified Eagle Medium (DMEM-HG; Life technology, NY, USA) consisting of 10% fetal bovine serum (Thermo, Logan, UT) and 1% penicillin–streptomycin solution (Corning, NY, USA). KG-1 cells were cultured in Iscove's Modified Dulbecco's Medium (IMDM; Life technology, NY, USA) consisting of 10% fetal bovine serum (Thermo, Logan, UT) and 1% penicillin–streptomycin solution (Corning, NY, USA). The cells were incubated at 37 °C and 5% CO_2_. When cells reached 80% confluence density, cells were separated by Accutase ® (Innovative Cell Technologies, SD, USA) and reseeded at a ratio 1:6.

### MTT cell viability assay

Macrophage cell line Raw264.7 and KG-1 cells were seeded 3 × 10^4^ cells per well in 96-well plates with culture medium for 24 h then supernatant was removed. Next cells were starved in serum-free culture for 16 h. After starvation, cells were treated with LPS (Sigma, Aldrich, USA) and IL-4 (PeproTech, NJ, USA) at different concentrations (LPS: 0–1.5 μg/mL, IL-4: 0–100 ng/mL) for 24 h. Following treatment, supernatant was removed and 1 mg/mL MTT (3-(4,5-Dimethylthiazol-2-yl)-2,5-diphenyltetrazolium bromide; USB, Cleveland, OH, USA) reagent was added to 96-well plates for 4 h incubation at 5% CO_2_ and 37 °C. Then the reagent was removed and crystal dissolved with dimethyl sulfoxide (DMSO; Bio Basic, YTO, CA) on the shaker for 10 min at 37 °C. The results were detected by using Multimode microplate readers Infinite 200 (TECAN, Switzerland) at a wavelength of 545 nm.

### Treatment of macrophages and IL-1β-hUCMSCs

Raw264.7 and KG-1 cells were plated 2 × 10^5^ cells in 30 mm plates with culture medium for 24 h. Then the supernatant was removed and cells were starved in serum-free medium for 16 h. After starvation, cells were treated with 0.0025, 0.5 and 1 μg/mL LPS or 5, 10 and 20 ng/mL IL-4 for 24 h. hUCMSCs were seeded 1 × 10^5^ cells in 60 mm petri dish with culture medium for 24 h and starved in serum-free DMEM-LG for 16 h. Afterward, cells were treated with 100 ng/mL human recombinant Interleukin-1β (IL-1β; PeproTech, NJ, USA) for 24 h.

### Co-culture system of macrophages and IL-1β-hUCMSCs

For direct co-culture, Raw264.7 and KG-1 cells were plated 2 × 10^5^ cells in 30 mm petri dishes with culture medium for 24 h and then starved in serum-free medium for 16 h. After starvation, cells were treated with 1 μg/mL LPS (polarization into M1 macrophages) and 20 ng/mL IL-4 (polarization into M2 macrophages) for 24 h. hUCMSc were seeded 1 × 10^5^ cells in 60 mm petri dishes for 24 h. Then starved in serum-free DMEM-LG medium for 16 h and treated with 100 ng/mL IL-1β for 24 h. After pre-treatment of macrophage cell lines and hUCMSCs, both were detached with Accutase ®and seeded into the same 30 mm well for co-culture.

For indirect co-culture, Raw264.7 and KG-1 cells were plated 2 × 10^5^ cells in 6 well plates with culture medium for 24 h and starved in serum-free medium for 16 h. After starvation, cells were treated with 1 μg/mL LPS (polarization into M1) or 20 ng/mL IL-4 (polarization into M2) for 24 h. hUCMSCs were seeded 1 × 10^5^ cells in 0.4 μm pore, 24 mm transwell inserts with culture medium for 24 h. Next, cells were starved in serum-free DMEM-LG for 16 h then treated with 100 ng/mL IL-1β for 24 h. After pre-treatment of macrophages and hUCMSCs, the transwell inserts with hUCMSCs were moved into 6 well plates to co-culture with Raw264.7 and KG-1 cells for 24 h.

### Western Blotting

Cells were washed with PBS and M-PER Mammalian Protein Extraction Reagent (Thermo, IL, USA) with 1% Halt Protease Inhibitor Cocktail (Thermo, IL, USA) was used to lysed cells. Extractions were vortexed for 1 min and centrifuged at 14,000×*g* for 10 min at 4 °C. Bio-Ray Protein Assay Dye Reagent (BIO-RAD, CA, USA) and multimode microplate readers (Infinite 200, TECAN) were used to determined protein concentrations. Protein samples (25 μg) were separated by 10% or 15% sodium dodecyl sulfate–polyacrylamide gel electrophoresis (SDS-PAGE) and transferred to Immun-Blot PVDF Membrane (BIO-RAD, CA, USA). Membranes were then blocked with 5% Fish Gelatin Blocking Buffer (AMRESCO, OH, USA) in Tris-buffered saline with tween 20 (TBST) on shaker for 1 h at room temperature (RT). Then membranes were washed with TBST for 5 min three times and incubated with caspase 3 antibody (GeneTex, CA, USA) diluted at 1:1000, caspase 8 antibody (GeneTex, CA, USA) diluted at 1:1000, caspase 9 antibody (GeneTex, CA, USA) diluted at 1:1000, DR4 antibody (GeneTex, CA, USA) diluted at 1:1000, DR5 antibody (Abcam, Boston, USA) diluted at 1:1000, iNOS antibody (Abcam, Boston, USA) diluted at 1:1000, TRAIL antibody (Cell Signaling, MA, USA) diluted at 1:1000 and beta-actin antibody (GeneTex, CA, USA) diluted at 1:10,000 in blocking buffer on shaker at 4 °C overnight. The membranes were washed with TBST for 5 min three times and incubated with Rabbit IgG antibody (HRP) (GeneTex, CA, USA) for 1 h on shaker at RT. The results were detected by enhanced chemiluminescence substrate (ECL) (T-Pro Biotechnology, New Taipei County, Taiwan) and LAS-4000 Luminescence Imaging System (GE, CT, USA). The quantification of the Western Blot was analyzed by AlphaEaseFC 4.0 software and the full-length Western blots was shown in Supplementary Fig. S1.

### Annexin V/PI apoptosis assay

After direct or indirect co-culturing with IL-1β-hUCMSCs, M1-polarized or M2-polarized macrophages were washed with PBS and detached by Accutase®. Then cell pellets were collected at 1200 rpm for 5 min and washed with PBS three times. The pellets were resuspended in 100 μL of 1 × Annexin V binding buffer and stained with 50 μg/mL Annexin V-FITC (Annexin V) and 100 μg/mL propidium iodide (PI) (Strong Biotech Corporation, TPE, ROC) for 15 min. After incubation, cells were washed with PBS twice. The results were analyzed immediately by flow cytometry (Beckman, IL, USA).

### Flow cytometry assay

KG-1, Raw264.7 cells or hUCMSCs were detached by Accutase®. Then cell pellets were collected at 1200 rpm for 5 min and washed twice with PBS. Afterward, cells were suspended in 100 μL PBS and labeled with different macrophages markers iNOS (Abcam, Boston, USA) and CD163 (BioLegend, CA, USA). hUCMSCs were labeled with CD105 (BioLegend, CA, USA). For the cell proliferation experiment, cells incubated were treated with 10uM BrdU for an hour then stained with BrdU antibody (Thermo, Logan, UT). After incubation of the antibody, cells were washed with PBS three times and analyzed immediately by flow cytometry (Beckman, IL, USA).

### Collagen-induced arthritis (CIA) mouse model and animal experiment

DBA/1J mice were purchased from BioLASCO Taiwan Co., Ltd. (Taipei, Taiwan) from Jackson Laboratory (Sacramento, CA) and maintained in the Laboratory Animal Center of National Yangming Chiao Tung University in 12 h dark/12 h light cycle with adequate food and water. The animal experimental procedures were approved by the Laboratory Animal Center of National Yangming Chiao Tung University (IACUC-1100334) and the reporting of animal experiments follows recommendations in the ARRIVE guidelines. Eight-week-old DBA/1J mice were used in this study (n = 24) and the data of dead mice were excluded from the subsequent analysis.

All the experiments were conducted according to ‘‘Protocol for the Successful Induction of Collagen-Induced Arthritis (CIA) in Mice’’ (Chondrex, Inc.). Eight-week-old DBA/1J mice were induced with type II collagen and Complete Freund's Adjuvant (CFA) by intradermal injection at the base of the tail to develop rheumatoid arthritis. Both male and female mice were randomly divided into four groups (Sham, RA, MSC, IL-1B MSC). Arthritis developed on day 35 after first injection, then, PBS, hUCMSCs or IL-1β-hUCMSCs were used for treatment by tail vein injection. Animals were anesthetized with Isoflurane on the indicated days and sacrificed by cervical detachment. The limbs of the mice were removed and handed over to Bio-Check Laboratories LTD (Taipei, Taiwan) to be embedded in wax and sectioned at a thickness of 3 μm.

### Immunohistochemistry assay

The sections of joints were deparaffinized with xylene and rehydrated using different percentages of ethanol. After rehydration, cells were washed with ddH2O to remove ethanol. Slides were blocked with endogenous peroxidase with 3% H_2_O_2_ for 15 min, then washed with PBST three times for 10 min. For antigen retrieval, the slides were placed in the glass staining tank and soaked in a sodium citrate buffer for 10 min at 95 °C. Then the glass staining tank was removed from the heater and the solution cooled down to room temperature (RT). The slides were washed with PBST three times for 10 min. After blocking with 5% Fish Gelatin Buffer (AMRESCO, OH, USA) and 0.1% triton X-100 in PBST (blocking Buffer) for 60 min at RT, the cells were then incubated with iNOS antibody (Abcam, Boston, USA) diluted at 1:100 and CD163 antibody (Abcam, Boston, USA) diluted at 1:100 at 4 °C overnight. The slides were washed with PBST for 10 min three times and incubated with Rabbit IgG antibody (HRP) (GeneTex, CA, USA) for one hour at RT. After incubation, cells were washed with PBST for 5 min three times, then the specimen was covered with DAB solution for 20 min. The slides were washed with H_2_O and counterstained with hematoxylin for 60 s. Finally, the slides were dehydrated with different concentrations of ethanol and ethanol was cleared with xylene. Then a mounting medium was used to mount coverslips over the specimen. The M1 and M2 macrophages of each group were counted in 3 different tissue sections. The cell counting results were analyzed and plotted with Prism 8.0 software.

### Statistical analysis

Quantitation data were represented as mean ± SEM. Statistical analysis data was analyzed by one-way ANOVA. P values < 0.05 were considered as statistically significant by Dunnett's test and Tukey's test. All quantitative analysis data were performed with Prism 6.0 software.

### Ethics approval and consent to participate

All experimental protocols were approved on March 11, 2021 by the Institutional Animal Care and Use Committee (IACUC) of National Yang Ming Chiao Tung University and were conducted in accordance with ethical regulations (Approval no. 1100334). The title of the approved project is “Effects of Interleukin Stimulated Human Umbilical Cord Mesenchymal Stem Cells on Macrophages for the Treatment of Rheumatoid Arthritis”.

## Supplementary Information

Supplementary Figure S1.

## Data Availability

All data needed to evaluate the conclusions in the paper are present in the paper.
