# Supplementary material for: The effects of IL-1β stimulated human umbilical cord mesenchymal stem cells on polarization and apoptosis of macrophages in rheumatoid arthritis
Source: Sci Rep. 2023 Jun 30;13:10612. doi: 10.1038/s41598-023-37741-6 (PMC10313744; doi:10.1038/s41598-023-37741-6)

The effects of IL-1β stimulated human umbilical cord mesenchymal stem cells on polarization and apoptosis of macrophages in rheumatoid arthritis

Ying-Xuan Zeng[1], Kuang-Yi Chou[2], Jeng-Jong Hwang[3] and Hwai-Shi Wang[1, *]

Supplementary Figure 1. Full- length Western blots.

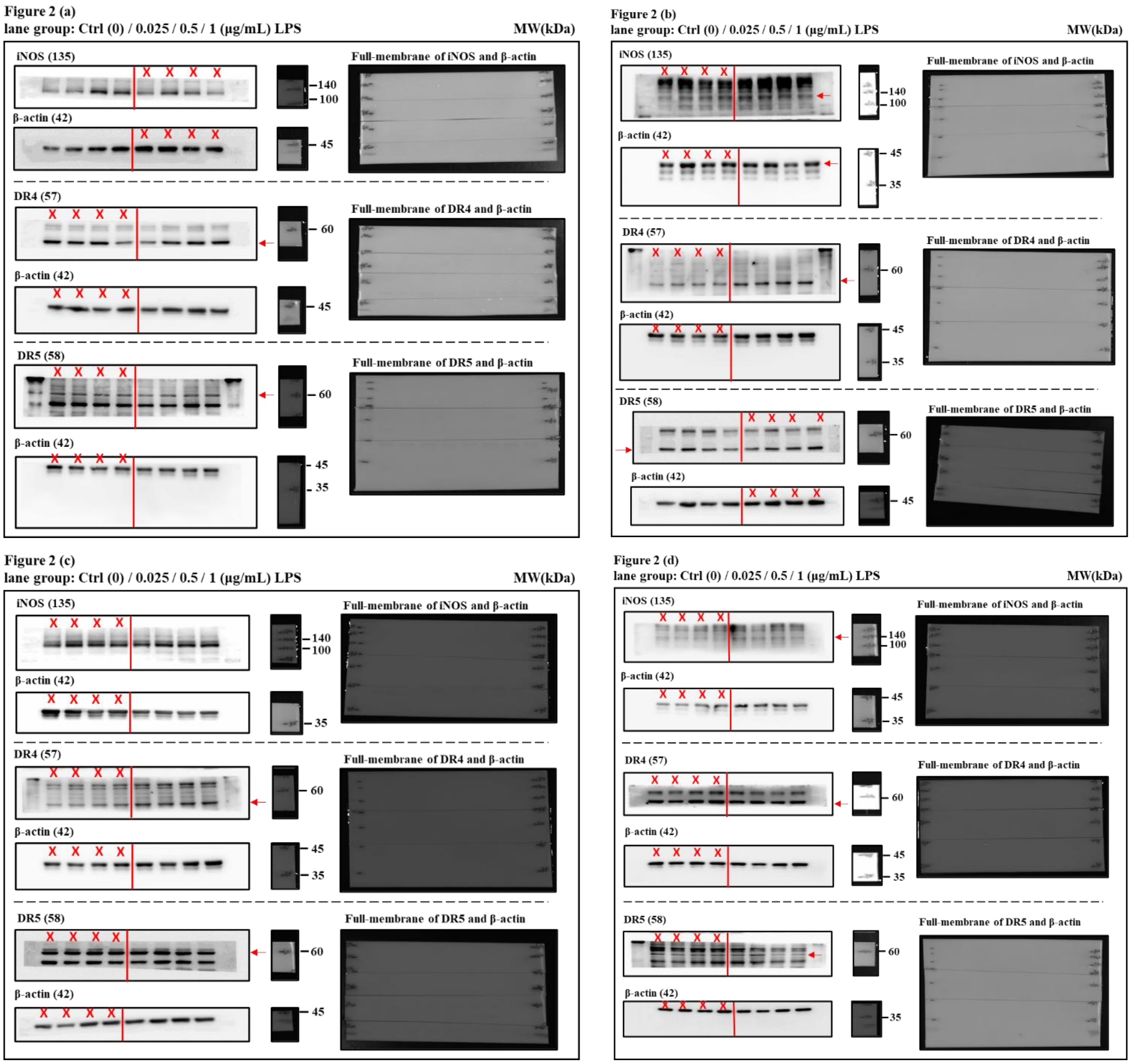

Figure 5 (a)

MW(kDa)

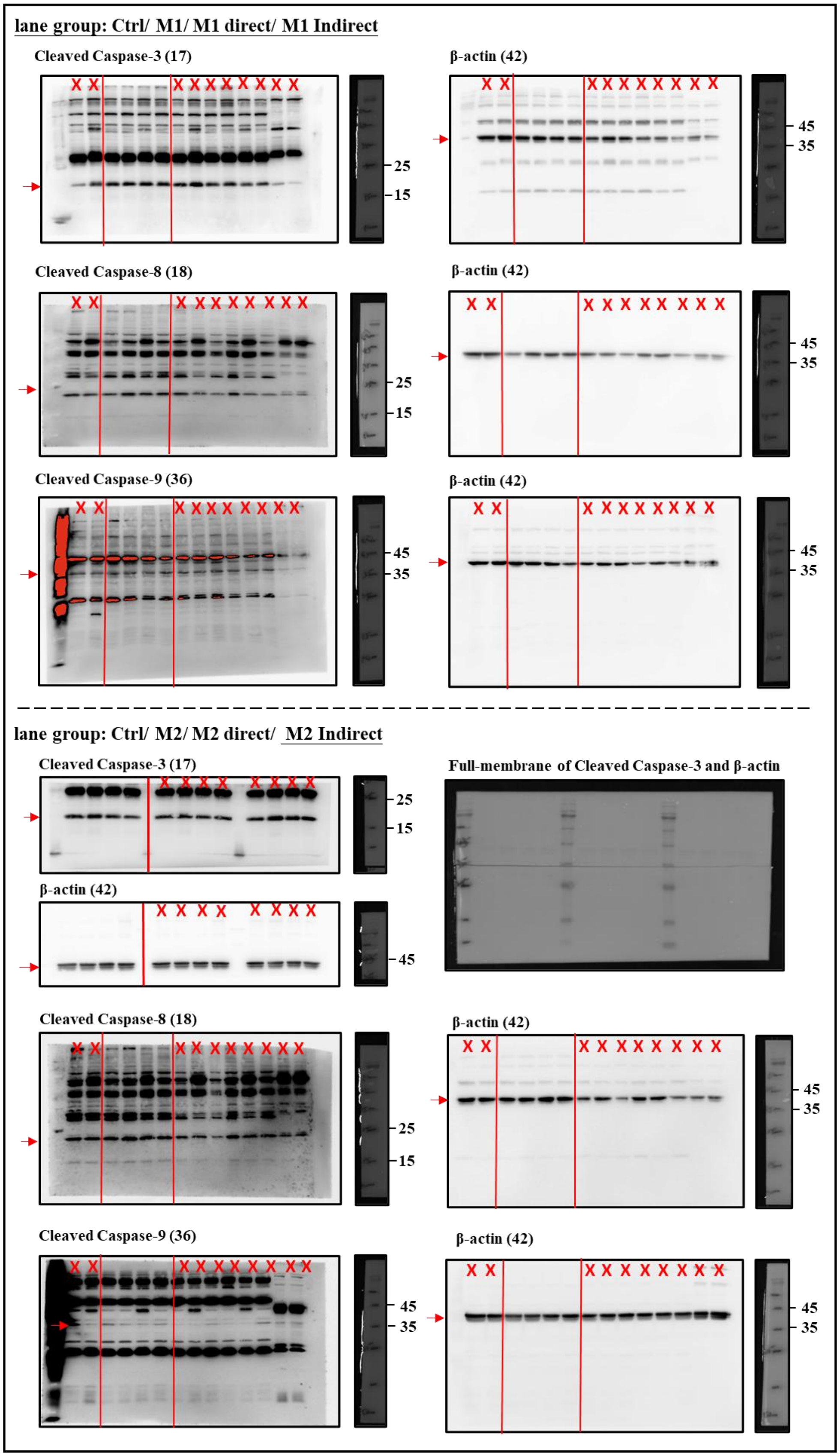

Figure 5 (b)

MW(kDa)

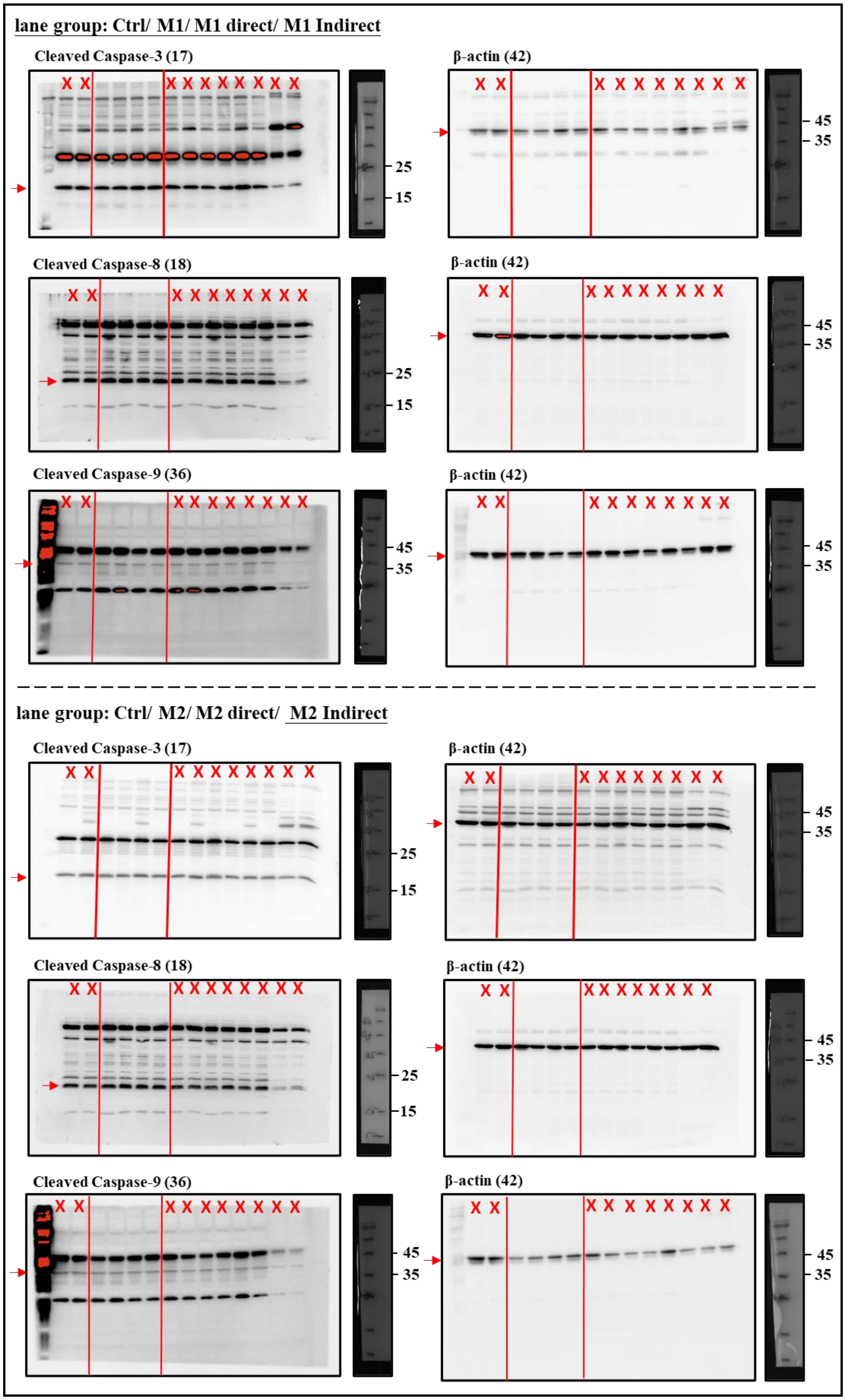

Supplement: Supplementary file 1 — Supplementary Figure S1. [file 41598_2023_37741_MOESM1_ESM.pdf]
